# Interaction between G-Quadruplex and Zinc Cationic Porphyrin: The Role of the Axial Water

**DOI:** 10.1038/s41598-017-11413-8

**Published:** 2017-09-08

**Authors:** Xiangzi Yao, Di Song, Tingxiao Qin, Chunfan Yang, Ze Yu, Xiaohong Li, Kunhui Liu, Hongmei Su

**Affiliations:** 1 Beijing National Laboratory for Molecular Sciences (BNLMS), Institute of Chemistry, Chinese Academy of Sciences, Beijing, 100190 China; 2 College of Chemistry, Beijing Normal University, Beijing, 100875 China; 3 University of Chinese Academy of Sciences, Beijing, 100049 China

## Abstract

The interaction of ligands with G-quadruplexes has attracted considerable attention due to its importance in molecular recognition and anticancer drugs design. Here, we utilize triplet excited state as a sensitive reporter to study the binding interaction of zinc cationic porphyrin (ZnTMPyP4) with three G-quadruplexes, AG_3_(T_2_AG_3_)_3_, (G_4_T_4_G_4_)2, and (TG_4_T)4. By monitoring the triplet decay dynamics of ZnTMPyP4 with transient absorption spectroscopy, the coexisted binding modes via π-π stacking of porphyrin macrocycle and the G-quartets are allowed to be identified quantitatively, which involve intercalation (25% and 36%) versus end-stacking (75% and 64%) for AG_3_(T_2_AG_3_)_3_ and (G_4_T_4_G_4_)2, and end-stacking (23%) versus partial intercalation (77%) for (TG_4_T)4. It is shown that the steric hindrance of the axial water decreases greatly the percentage of intercalation. Further, a rapid assessment of binding stoichiometry is fulfilled by measuring the triplet decay dynamics under various [G-quadruplex]/[ZnTMPyP4] ratios. The binding stoichiometric ratios of G-quadruplex/ZnTMPyP4 are 1:2 for AG_3_(T_2_AG_3_)_3_, 1:1 for (G_4_T_4_G_4_)2, and 1:2 for (TG_4_T)4, which agree well with results obtained by the conventional method of continuous variation analysis. These results reveal a clear scenario of G-quadruplex/ZnTMPyP4 interaction and provide mechanistic insights for the application of anticancer drug designs using G-quadruplex as target.

## Introduction

The single-stranded telomere segment at the 3′ ends of chromosomal DNA is believed to form a variety of four-stranded structures known as G-quadruplexes, based on a G-tetrad structure of four Hoogsteen-paired and coplanar guanines^1^. The unique structure of G-quadruplex has an ability to inhibit the activity of telomerase, a ribonucleoprotein enzyme that is usually activated in 85–90% of cancer cells^2^. The potential application of G-quadruplexes to cancer treatment has propelled investigation of small molecules (ligands) that promote and/or stabilize G-quadruplex formation. A large number of ligands, displaying varying degrees of affinity and ability to stabilize G-quadruplexes, have been identified or synthesized successively^3–6^. Among these G-quadruplex ligands, the free-base porphyrin (5, 10, 15, 20-tetrakis (1-methyl-4-pyridyl)-21H, 23H-porphine) (TMPyP4) belongs to a planar aromatic system containing heteroatoms, and can thus bind to G-quadruplexes through π-π stacking^6–11^. Insertion of a metal ion into this porphyrin allows the modulation of its binding capacity toward G-quadruplexes DNA. It is shown that some metalloporphyrins can produce stronger π-interactions with G-quartets, enabling them to be a more attractive candidate for anticancer drug studies^12–18^. However, compared with intensive reports for interactions of TMPyP4 with G-quadruplexes, studies on the G-quadruplexes interaction with metalloporphyrins, especially for high coordination metal complexes, are limited at present. On the other hand, various metal ions afford variation of the charge and axial coordination in the center of the porphyrin, which may lead to different G-quadruplexes binding properties^16–20^. Thus, a full understanding for the interaction between metalloporphyrin and G-quadruplex seems to require case-by-case studies.

**Figure 1:**
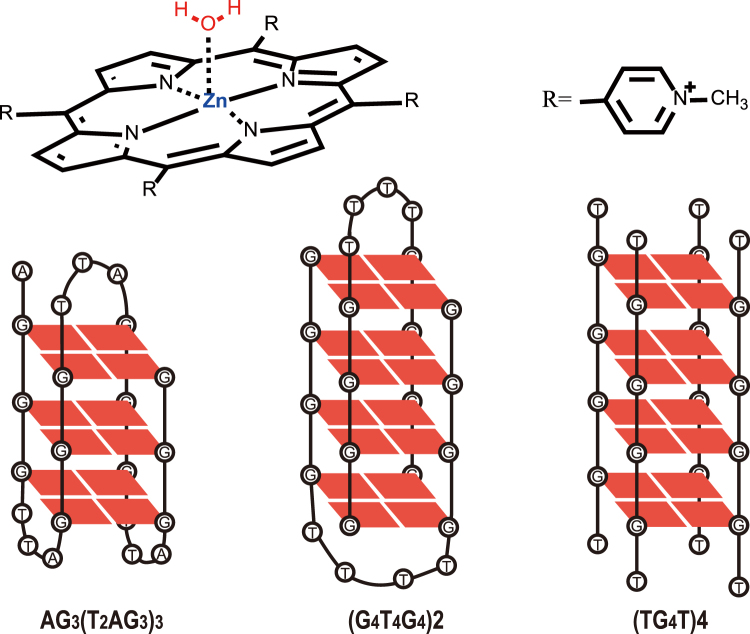
The Structure of ZnTMPyP4 and schematic topology diagrams for the three G-quadruplexes: AG_3_(T_2_AG_3_)_3_ (in Na^+^ containing buffer), (G_2_T_4_G_4_)2 and (TG_4_T)4.

ZnTMPyP4 belongs to the metalloporphyrin family and attracts research interests because it is a typical penta-coordination system for the study of G-quadruplexes/ligand interaction^16–21^. As a derivative of TMPyP4, ZnTMPyP4 is found to maintain the similar size to TMPyP4^21^. Noticeably, ZnTMPyP4 has a coordinated axial water molecule perpendicular to the aromatic plane, thus adopts square pyramidal geometry, which is different from its parent molecule TMPyP4 (Fig. 1) and may result in distinct binding behavior between G-quadruplexes and ZnTMPyP4^21^. Interestingly, the end-stacking of ZnTMPyP4 onto the terminal G-quartets was inferred from the bisignate peak in the induced circular dichroism (ICD) spectra^22^. Although the putative steric hindrance of the axial water of ZnTMPyP4 may prevent the intercalation binding between two adjacent G-quartets, it was ever characterized that ZnTMPyP4 can intercalate between G:C base pairs of the duplex DNA [poly(dG-dC)]_2_, where the axial water of ZnTMPyP4 was assumed to be released by gaining enough energies during its intercalation process^23^. It follows that the axial water of ZnTMPyP4 invokes confusion for understanding the binding modes of ZnTMPyP4 with G-quadruplexes.

In this context, the present work attempts to examine the binding interactions of G-quadruplex/ZnTMPyP4 and clarify the role of the axial water. Three G-quadruplexes, intramolecular single-stranded AG_3_(T_2_AG_3_)_3_, intermolecular double-stranded (G_4_T_4_G_4_)2, and intermolecular four-stranded (TG_4_T)4 (Fig. 1) are systematically selected as the G-quadruplex target models due to the following reasons: (1) From structural considerations, there is at least a wide groove (about 10 Å across) in the structures of AG_3_(T_2_AG_3_)_3_ and (G_4_T_4_G_4_)2^24, 25^, thus allowing porphyrin-sized ligands (the pyridyl N to pyridyl N distance is about 9 Å)^26^ to fully get into the groove and further intercalate between two neighboring G-quartets; While for (TG_4_T)4, four relative narrow grooves (about 6.6 Å across)^27^ cannot offer sufficient room to accommodate porphyrin-sized ligands by stacking with G-quartets plane, which may prohibit the intercalation binding; (2) Interactions between the three G-quadruplexes and TMPyP4 have been extensively investigated^6–11^, which can provide abundant data to be compared with the results of current work. Such comparison would be beneficial for examining the effect of the axial water of ZnTMPyP4 on the binding interactions.

The transient spectral method utilizing triplet excited state of ligands as sensitive reporters is a powerful tool to recognize multiplex G-quadruplex/ligand interactions in a single assay^28^. Some G-quadruplex ligands with large and planar aromatic ring have high quantum yields of triplet states upon photo excitation, and their triplet lifetimes are highly sensitive to the local bound environment that prevents molecular oxygen access and triplet quenching, thus making it possible to distinguish different binding sites and assess contributions of coexisted binding modes. The method has been used to probe coexisted binding modes for interactions of G-quadruplexes/TMPyP4, obtaining crucial information that could reconcile controversial binding modes^28^. Notably, structural features of ZnTMPyP4 with large aromatic rings and diamagnetic metal ion Zn^2+^, result in high quantum yields of triplet states^29^, which provides an opportunity to use the triplet reporter method for investigating its binding to the three G-quadruplexes. Here in this work, the transient absorption spectroscopy measurements for the triplet state ZnTMPyP4 bound with G-quadruplex show biexponential decay dynamics, with a slower lifetime component and a faster lifetime component markedly longer than that of free ZnTMPyP4. Two coexisted binding modes are thus revealed and their respective contributions are also quantitatively estimated. Compared with G-quadruplex binding results of TMPyP4^28^, the contributions of intercalation mode (the slower lifetime component) are significantly decreased, indicating that the steric hindrance of axial water is crucial for affecting the G-quadruplex-binding of ZnTMPyP4. Extensive study aimed at a rapid assessment of binding stoichiometry is conducted further by measuring the triplet state decay dynamics of ZnTMPyP4 under various molar ratios of [G-quadruplex]/[ZnTMPyP4]. These results reveal a clear scenario of G-quadruplex/ZnTMPyP4 interactions (the coexisted binding modes, the percentage of each binding mode, and the role of the axial water), providing important insights for the rational design of G-quadruplex ligands in cancer therapy.

## Results and Discussion

### Characterizing G-Quadruplex DNA structures and G-quadruplex/ligand binding

On the basis of previous work^30–32^, we dissolved the oligonucleotides TG_4_T in K^+^ buffer to form the structures of parallel four-stranded G-quadruplexes, and dissolved AG_3_(T_2_AG_3_)_3_ and G_4_T_4_G_4_ in Na^+^ buffer to form the antiparallel basket-type and dimer-hairpin-folded antiparallel G-quadruplexes respectively. These G-quadruplex structures were further verified by using circular dichroism (CD) spectroscopy, where parallel G-quadruplexes usually display a distinct positive CD signal at about 263 nm and the CD signal for antiparallel structure is at approximately 295 nm. The measured CD spectra (Figure S1) for AG_3_(T_2_AG_3_)_3_ and (G_4_T_4_G_4_)2 are featured with a positive peak at 295 nm and a negative peak at 263 nm, while the CD spectrum of (TG_4_T)4 exhibits a positive peak at 263 nm and a negative peak at 244 nm^33^. These CD results confirm the targeted conformation patterns shown in Fig. 1.

**Figure 2:**
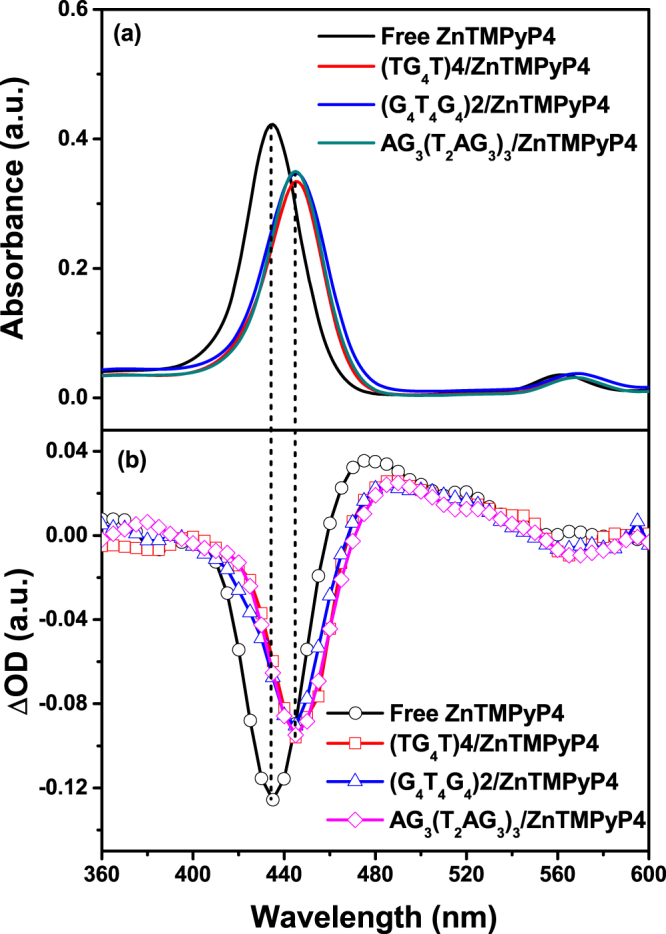
(**a**) Steady-state UV-vis absorption spectra, for free ZnTMPyP4 (2 μM) and its complexes with each of the three G-quadruplex (12 μM) in buffer solution (10 mM Tris-HCl, 1 mM EDTA, and 100 mM KCl or NaCl, pH = 7.5). (**b**) Transient absorption spectra instantaneously (50 ns) after laser flash photolysis upon 355 nm excitation, for free ZnTMPyP4 (2 μM) and its complexes with each of the three G-quadruplexes (12 μM) in buffer solution (10 mM Tris-HCl, 1 mM EDTA, and 100 mM KCl or NaCl, pH = 7.5).

Figure 2(a) shows the UV-vis absorption spectra of three G-quadruplex/ZnTMPyP4 mixtures and free ZnTMPyP4 (molar ratio of 6:1). Compared with free ZnTMPyP4, red shifts from 435 nm to ~445 nm and hypochromicity degrees of ~15% at the Soret band maxima are observed for the three G-quadruplex/ZnTMPyP4 mixtures. These two characteristics of the absorption spectra suggest the existence of G-quadruplex/ZnTMPyP4 interactions^17^. To further confirm G-quadruplex/ZnTMPyP4 interactions, we estimated binding constants for ZnTMPyP4 with AG_3_(T_2_AG_3_)_3_, (G2T4G4)2, (TG4T)4 by using UV absorbance data at different [G-quadruplex/[ZnTMPyP4] molar ratios and a previously proposed binding model^34, 35^, which are 3.6 × 10^6^ M^−1^, 1.0 × 10^5^ M^−1^ and 1.0 × 10^6^ M^−1^, respectively. These results show that strong interactions exist between ZnTMPyP4 and the three G-quadruplexes and no free ligand will be present in solution.

### Differentiating the binding modes by triplet decay dynamics

In order to further reveal the interaction modes, transient absorption spectroscopy measurements of the triplet ZnTMPyP4 were performed in the absence and presence of the G-quadruplex. The G-quadruplex/ZnTMPyP4 molar ratio of 6:1 was employed for ensuring that all the ZnTMPyP4 molecules are bound to G-quadruplexes and avoiding the population of free ZnTMPyP4 in bulk. After laser flash photolysis upon 355 nm excitation, the transient UV-vis absorption spectra of free ZnTMPyP4 and the three G-quadruplexes/ZnTMPyP4 complexes were obtained, as shown in Fig. 2(b). In the transient spectrum of free ZnTMPyP4, a positive peak at 480 nm and a negative peak at 430 nm are observed, corresponding to triplet excited state formation and ground state depletion separately. In the transient spectra of G-quadruplex/ZnTMPyP4 complexes, the positive peaks and negative peaks shift about 10 nm to the red, accompanied with different degrees of hypochromicity. Therefore, the following excited state dynamics measurements were carried out for free ZnTMPyP4 at 480 nm and for G-quadruplex/ZnTMPyP4 complexes at 490 nm.

**Figure 3:**
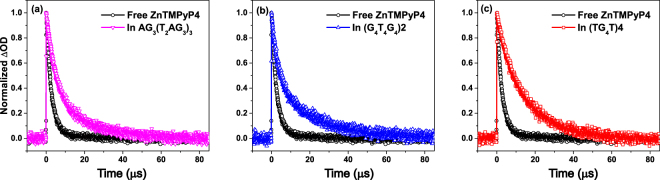
Normalized triplet decay signals after laser flash photolysis of ZnTMPyP4 (2 μM) upon 355 nm excitation in the absence (black) and presence of each G-quadruplex (12 μM): (**a**) AG_3_(T_2_AG_3_)_3_, (**b**) (G_4_T_4_G_4_)2, (**c**) (TG_4_T)4. Fitted curves are shown by solid lines.

Figure 3 presents the triplet decay traces for ZnTMPyP4 respectively bound with each of three G-quadruplexes, in comparison with free ZnTMPyP4. Table 1 summarizes their respective fitted lifetime values. For free ZnTMPyP4, it is observed that its triplet state decay follows a mono-exponential law with a lifetime of 2.9 ± 0.02 μs, in agreement with literature results^23^. In the presence of G-quadruplexes, the triplet state decay of ZnTMPyP4 becomes much slower and follows a second order exponential law with a fast (τ_1_) and a slow (τ_2_) lifetime component. As illustrated in Table 1, the fast lifetime components (τ_1_) are 5.8 ± 0.1 μs, 5.2 ± 0.2 μs, and 5.3 ± 0.1 μs, and the slow lifetime values (τ_2_) are 32.5 ± 0.6 μs, 30.7 ± 0.9 μs, and 17.3 ± 0.2 μs, for ZnTMPyP4 in complex with AG_3_(T_2_AG_3_)_3_, (G_4_T_4_G_4_)2, and (TG_4_T)4, respectively. Since the triplet state lifetime of the bound ligand reflects the degree of shielding of the triplet ligand from oxygen quenching, the biexponental triplet decay for the bound ZnTMPyP4 strongly implies the simultaneous existence of two binding modes.

**Table 1:** Triplet decay lifetimes of ZnTMPyP4 and TMPyP4 in air-saturated solution and in their complexes with G-quadruplexes, obtained from biexponential fitting (*I* = *I*_0_ + *A*_*1*_ *e* ^−*t/τ1*^ + *A*_*2*_ *e* ^−*t/τ2*^). Pre-exponential factors for the two lifetime components yield the respective percentages of two binding modes (values shown in brackets).

Sample	τ_1_ (μs)	τ_2_ (μs)
ZnTMPyP4	2.9 ± 0.02	
ZnTMPyP4-AG_3_(T_2_AG_3_)_3_	5.8 ± 0.1 (75%)	32.5 ± 0.6 (25%)
ZnTMPyP4-(G_4_T_4_G_4_)2	5.2 ± 0.2 (64%)	30.7 ± 0.9 (36%)
ZnTMPyP4-(TG_4_T)4	5.3 ± 0.1 (23%)	17.3 ± 0.2 (77%)
TMPyP4	1.6 ± 0.02*	
TMPyP4-AG_3_(T_2_AG_3_)_3_	3.9 ± 0.1 (62%)*	29.0 ± 0.9 (38%)*
TMPyP4-(G_4_T_4_G_4_)2	3.6 ± 0.1 (17%)*	28.0 ± 0.6 (83%)*
TMPyP4-(TG_4_T)4	3.2 ± 0.1 (29%)*	12.2 ± 0.5 (71%)*

^*^The data are cited from ref. 28.

Previous studies have shown that, among all possible binding modes, intercalation protects the ligand from oxygen access the most, and the triplet lifetime of intercalated component should thus be the longest^23, 28, 36^. For example, a triplet lifetime of TMPyP4 (~28 μs) was measured for the intercalated population in G-quadruplexes, which is much longer than that of the population bound in end-stacking mode (~3 μs)^28^. In this work, for AG_3_(T_2_AG_3_)_3_ and (G_4_T_4_G_4_)2, the two longest lifetime components of 32.5 ± 0.6 μs and 30.7 ± 0.9 μs are comparable with intercalative lifetime (~35 μs) of ZnTMPyP4 in [poly(dG-dC)]_2_^23^ and thus should correspond to the intercalation of ZnTMPyP4 between two adjacent G-quartets (Figure S2(a) and (b)). It is noted that such a slow lifetime (32.5 ± 0.6 μs or 30.7 ± 0.9 μs) component is not observed in the triplet decay of the binding of ZnTMPyP4 with (TG_4_T)4 (Table 1). This agrees with the G-quadruplex structural features: the groove widths of about 10 Å^26^ for AG_3_(T_2_AG_3_)_3_ and (G_4_T_4_G_4_)2 can permit ZnTMPyP4 to intercalate and stack within two adjacent G-quartets, but grooves of (TG_4_T)4 (about 6.6 Å across)^27^ are too narrow to allow the intercalation. The disappearance of the triplet lifetime ~30 μs for (TG_4_T)4 further confirms the assignment for the intercalation mode of ZnTMPyP4 with AG_3_(T_2_AG_3_)_3_ and (G_4_T_4_G_4_)2, as a structural control.

The fast lifetime components, 5.8 ± 0.1 μs, 5.2 ± 0.2 μs, and 5.3 ± 0.1 μs are almost identical for ZnTMPyP4 in complex with the three G-quadruplexes, meaning that these lifetime components may correspond to the same interaction mode. The short lifetime values also mean that the interaction mode should involve a rather low shielding from oxygen. In our previous work dealing with triplet decay dynamics of TMPyP4 in four G-quadruplexes, fast lifetime components (~3 μs) are assigned to the population of end-stacking binding mode onto the terminal G-quartet within loop regions. The end-stacking interaction mode provides a limited protection from triplet quenching, resulting in only a two-fold increase of triplet state lifetime from ~1.6 μs (free TMPyP4) to ~3 μs^28^. Here for ZnTMPyP4, the fast lifetime components, 5.8 ± 0.1 μs, 5.2 ± 0.2 μs, and 5.3 ± 0.1 μs are also about two times longer than the lifetime of free ZnTMPyP4. This is indicative of similar degree of shielding of the triplet ligand from oxygen quenching. Therefore, these fast lifetime components can be also ascribed to end-stacking of ZnTMPyP4 onto the terminal G-quartets, as shown in Figure S2(a)–(c). In the geometries associated with this binding mode, one side of pophyrin macrocycle faces the terminal G-quartet, while the other side faces various loops (TTA loop for AG_3_(T_2_AG_3_)_3_, TTTT loop for (G_4_T_4_G_4_)2) or the thymine bases at 3′ or 5′ ends. It is known that the bases located in the loop region or at 3′ (5′) ends of DNA strands have large freedoms and can even extend far away from the G-quartets due to little π-π stacking interaction with G-quartet. For example, the lateral loop of AG_3_(T_2_AG_3_)_3_ extends up to 10 Å from the core G-quartets^25^. It follows that these loops and the thymine bases at 3′ or 5′ ends can only offer very limited protection from oxygen access compared with the G-quartet plane. Therefore, the end-stacking binding mode corresponds to the faster lifetime component (~5 μs for ZnTMPyP4 in this work). Thus, our results also confirm the end-stacking mode, which is in accordance with previous studies of resonance energy transfer and ICD that indicated the end-stacking interactions for ZnTMPyP4 with AG_3_(T_2_AG_3_)_3_^22^.

**Figure 4:**
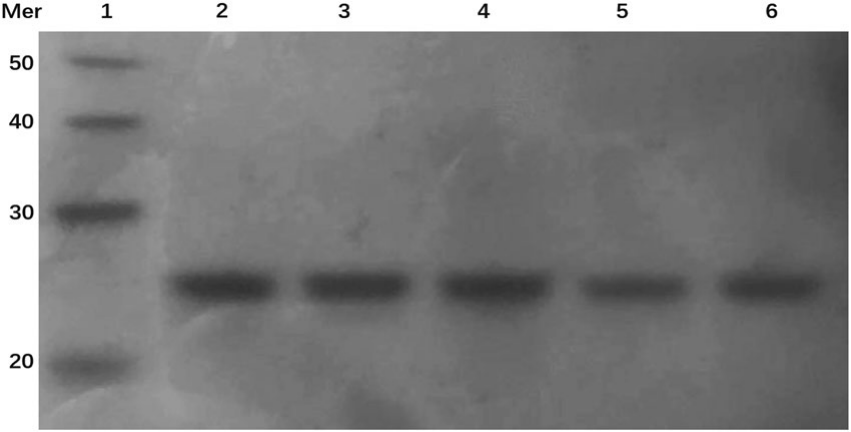
The polyacrylamide gel analysis of (TG_4_T)4 in the presence of K^+^. Lane 1: DNA marker 10-mer ladder; Lane 2: 1.5 μM (TG_4_T)4; Lane 3: 1.5 μM (TG_4_T)4 with 0.25 μM TMPyP4; Lane 4: 1.5 μM (TG_4_T)4 with 0.25 μM ZnTMPyP4; Lane 5:1.5 μM (TG_4_T)4 with 3 μM TMPyP4; and Lane 6: 1.5 μM (TG_4_T)4 with 3 μM TMPyP4. Full-length gel is presented in Figure S3.

As for the lifetime component of 17.3 ± 0.2 μs for the binding of ZnTMPyP4 with (TG_4_T)4, it is much longer than the typical end-stacking lifetime (~5 μs), and much shorter than the typical intercalative lifetime (~31 μs). Obviously, the lifetime component cannot be assigned to either end-stacking or intercalation, but should correspond to a binding mode that can offer a moderate degree of protection for ligands. In our previous work, a similar lifetime component of 12.2 ± 0.5 μs was observed in (TG_4_T)4/TMPyP4 complex and was tentatively assigned to the sandwich-type binding mode^28^. Is the lifetime component of 17.3 ± 0.2 μs for ZnTMPyP4 ascribed to the sandwich-type binding mode? If the hypothesis is true, a G-quadruplex dimer structure will form by vertical stacking of two (TG_4_T)4, where ZnTMPyP4 is sandwiched between the terminal tetrads of two (TG_4_T)4 monomers (Figure S2(c)). To scrutinize this possibility, an additional native polyacrylamide gel electrophoresis (PAGE) was carried out to determine whether there is the formation of dimeric G-quadruplex and the sandwich-type binding mode. As shown in Fig. 4, 24-mer (TG_4_T)4 exhibits a sharp band (lane 2) whose migration rate is between those of the 20-mer ssDNA marker and 30-mer ssDNA marker (lane 1), which means that (TG_4_T)4 G-quadruplex is only in monomeric form. If the 48-mer dimeric G-quadruplex forms, a migration rate that is very close to that of the 50-mer ssDNA marker should be observed in gel electrophoresis photograph. When porphyrin binds with (TG_4_T)4, the (TG_4_T)4/TMPyP4 complex (lane 3 and lane 5) and (TG_4_T)4/ZnTMPyP4 complex (lane 4 and lane 6) both migrate in the same speed as that of the monomeric (TG_4_T)4, indicating that the dimeric G-quadruplexes are not formed either. Therefore, it can be concluded that the two intermediate lifetimes cannot be assigned to the sandwich-type binding mode for the (TG_4_T)4/ZnTMPyP4 and (TG_4_T)4/TMPyP4 complexes.

Considering the structural feature of (TG_4_T)4, the most likely binding mode for the two intermediate lifetimes should be the partial intercalation (Figure S2(c)). As discussed above, (TG_4_T)4 has four equal grooves but the groove widths (about 6.6 Å across) are too narrow to allow porphyrin-sized ligands to fully get into the groove and further intercalate within two neighboring G-quartets, compared to the other two G-quadruplexes^27^. In this case, porphyrin-sized ligands could only partially intercalate into the grooves of (TG_4_T)4, which results in only a partial screening from oxygen quenching.

Additionally, we measured the triplet decay dynamics of ZnTMPyP4 in three G-quadruplexes at different ionic strengths (I = 0.01–0.3). As shown in Figure S4, the triplet decay rates of ZnTMPyP4 were accelerated with the increase of ionic strengths. For ZnTMPyP4 interacting with (G_4_T_4_G_4_)2, the acceleration of triplet decay is the most obvious. Furthermore, when the ionic strength increases to I = 0.2, a third lifetime component (3.4 ± 0.1 μs) is required to be included in addition to the lifetime components of 5.8 ± 0.1 μs and 30.3 ± 0.5 μs for obtaining a good fitting for the kinetics traces. For ZnTMPyP4 interacting with AG_3_(T_2_AG_3_)_3_ when the ionic strength increases to I = 0.3, three lifetime values (3.4 ± 0.1 μs, 5.8 ± 0.2 μs, and 32.0 ± 0.9 μs) are also necessary for the decay fitting. Even for the decay of ZnTMPyP4 in (TG_4_T)4 with the smallest alteration, it also requires three exponential law with the lifetimes of 3.3 ± 0.1 μs, 5.1 ± 0.1 μs, and 18.8 ± 0.3 μs at the high ionic strength I = 0.3. Noticeably, the third lifetime components ~3.4 μs newly involved are only 17% longer than free ZnTMPyP4 (2.9 μs). We believe that these very fast lifetime components most likely correspond to the electrostatic interaction mode, because they agree well with the fast lifetime component (3.5 μs) of ZnTMPyP4 in [poly(dG-dC)]_2_, which had been assigned as the electrostatic interaction binding mode by Chirvony, V. S. *et al* ^23^. Considering that a buffer with high ionic strength can hinder porphyrin-sized ligands from getting access to DNA molecules, e.g. duplex, tetraplex^23, 37–39^, it is reasonable that a portion of ZnTMPyP4 molecules are bound electrostatically by the charged pyridyl groups to the outside of G-quadruplexes (i.e., to the phosphate backbone) with a minimal interaction between ZnTMPyP4 macrocycle and G-quadruplexes. Therefore, these control experiments at high ionic strength exclude the possibility that the short lifetime components (~5.3 μs) are caused by the electrostatic interaction.

**Figure 5:**
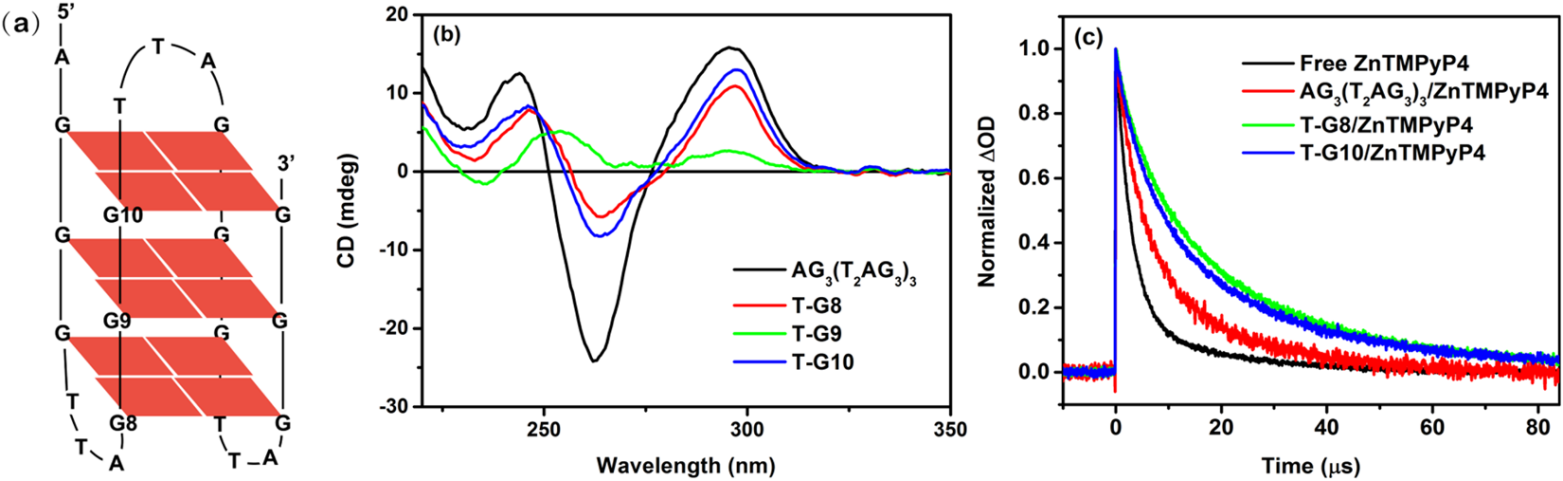
(**a**) The G-quadruplex AG_3_(T_2_AG_3_)_3_ for mismatch control: the thymine (T) substitutions of G base in the positions of G8, G9 and G10, respectively. (**b**) CD spectra of normal AG_3_(T_2_AG_3_)_3_ (10 μM) and the mismatched AG_3_(T_2_AG_3_)_3_ G-quadruplexes (10 μM): T-G8, T-G9, and T-G10. (**c**) Normalized triplet decay signals after laser flash photolysis of ZnTMPyP4 (2 μM) upon 355 nm excitation in the presence of normal AG_3_(T_2_AG_3_)_3_ and mismatched AG_3_(T_2_AG_3_)_3_ G-quadruplexes T-G8 and T-G10.

To reinforce the assignment of the intercalation mode, we measured the triplet decay of ZnTMPyP4 in the mismatched AG_3_(T_2_AG_3_)_3_ G-quadruplex, where a thymine (T) base separately replaces G8, G9 and G10 bases to introduce a direct perturbation of the π-stacking along G-quartets as shown in Fig. 5(a). The CD spectra (Fig. 5(b)) show that the T-substitution of G9 (denoted as T-G9) leads to an obvious structural change, indicating that the integrity of the core G9-quartet is the key to sustain the overall structure. In comparison, the other two mismatched G-quadruplexes (T-G8 and T-G10) maintain the typical feature of antiparallel structure as of the normal AG_3_(T_2_AG_3_)_3_. Consequently, we performed control experiments for ZnTMPyP4 interacting with the T-G8 and T-G10 mismatched G-quadruplex that still have well-defined structures. Since the replacement of T base tends to weaken the π-π stacking and increase the spacing between the mismatched and the adjacent normal G-quartets, it is expected that the intercalation mode corresponding to the slow lifetime component could be facilitated and the percentage of intercalation population should be increased. In this case, the triplet decay of ZnTMPyP4 would be decelerated. Indeed, it is observed in the kinetics traces (Fig. 5(c)) that the triplet decay rates of ZnTMPyP4 in the T-G8 and T-G10 mismatched G-quadruplexes are both slowed down markedly, compared with that in AG_3_(T_2_AG_3_)_3_. Meanwhile, kinetics fitting (discussed in the next section) shows that the percentage of the intercalation mode is increased to 63% for T-G8 and 53% for T-G10, compared with the value of 25% for AG_3_(T_2_AG_3_)_3_. In accordance with the increase of the intercalation percentage, the end-stacking binding mode populations are decreased. This is probably caused by fact that the T-G8 and T-G10 mismatch both introduce perturbations for the terminal G-quartets such that the end-stacking binding is not favored in this case. Whereas for intercalation, it is largely affected by the core G9-quartet that still maintains the normal π-stacking and the T-G8 or T-G10 mismatch only increase the inter-quartet spacing, leading to increased probability of intercalation. These results measured for the mismatch control demonstrate further that the ~30 μs slow lifetime components are most likely ascribed to the intercalation binding mode. Other structural evidence to support the existence of the intercalation binding would be highly desirable, for which we hope this work could arouse research interests of relevant structural studies such as nuclear magnetic resonance (NMR).

### Examining the role of the axial water of ZnTMPyP4

The structure of ZnTMPyP4 is similar to that of TMPyP4 except the difference of its axially-coordinated water, thus, comparisons of their G-quadruplex-binding behavior can reveal the role of axial water of ZnTMPyP4. Nevertheless, previous studies show that both the intercalation mode and end-stacking mode are observed for G-quadruplex-binding with TMPyP4, which is identical to G-quadruplex-binding with ZnTMPyP4^11, 28^. In this case, it is difficult to estimate the effect of the axial water simply based on qualitative results. We thus turn to quantitative measurements based on the triplet reporter method, which can not only recognize different binding modes, but also can estimate contributions of each coexisted binding mode that could not possibly be obtained by conventional techniques.

Subject to identical laser excitation, the ZnTMPyP4 molecules bound in different environment are supposedly equal in the transient response. Consequently, the contributions of each coexisted binding mode can be obtained by pre-exponential factors of the two lifetime components in the second order exponential fitting equation. Table 1 lists these fitting values. First, for the binding of TMPyP4 with (TG_4_T)4, it is shown that the end-stacking binding mode accounts for 29%, the partial intercalation binding mode accounts for 71%^28^. When TMPyP4 is replaced by ZnTMPyP4, the percentages of these two binding modes are 23% and 77%, which hardly alters. This indicates that the effect of the axial water on non-intercalative mode is insignificant. Second, for the binding of TMPyP4 with AG_3_(T_2_AG_3_)_3_, end-stacking is the preferred binding mode (62%), while intercalation accounts for 38%^28^. When TMPyP4 is replaced by ZnTMPyP4, the end-stacking increases to 75%, and the intercalation decreases to 25%. For the binding of TMPyP4 with (G_4_T_4_G_4_)2, intercalation is the predominant binding mode with the proportion of 83%^28^. When ZnTMPyP4 is bound with (G_4_T_4_G_4_)2, intercalation is markedly decreased to 36% and the end-stacking becomes dominant (64%). These data clearly indicate that the axial water hinders ZnTMPyP4 from intercalating between two neighboring G-quartets. It is noticeable that (G_4_T_4_G_4_)2 experiences a more significant binding mode pattern change than AG_3_(T_2_AG_3_)_3_ after TMPyP4 is replaced with ZnTMPyP4. This may be ascribed to the stronger effect of the axial water on intercalation and the originally preferred binding mode being intercalation for (G_4_T_4_G_4_)2 interacting with TMPyP4.

Meanwhile, these data can also rationalize why the intercalation mode was not previously characterized by conventional methods. In fact, the steady-state measurements can usually reflect the feature of the main binding site while the feature of the minor binding mode may be hidden. For AG_3_(T_2_AG_3_)_3_/ZnTMPyP4 complex, ICD was featured with the dominant end-stacking mode by a bisignate peak^22^. On the basis of such spectral features, the end-stacking was readily determined while the intercalation with a small proportion was not revealed. Here by monitoring the triplet decay dynamics, we show that the transient spectral method can identify the coexisted binding modes and obtain quantitative knowledge of percentage of different binding modes, thus providing a complete picture for interaction modes between G-quadruplexes and ZnTMPyP4.

### Determining the binding stoichiometry

**Figure 6:**
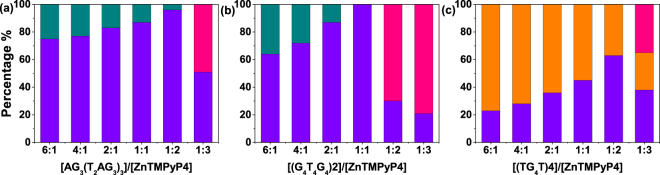
Histogram plots for the population percentage of free ZnTMPyP4 (pink), ZnTMPyP4 bound in the intercalation mode (dark cyan), ZnTMPyP4 bound in the end-stacking mode (violet), and ZnTMPyP4 bound in the partial intercalation mode (orange), obtained for different [G-quadruplex]/[ZnTMPyP4] molar ratios of 6:1, 4:1, 2:1, 1:1, 1:2 and 1:3.

The binding stoichiometry is a significant factor to understand binding mechanism of G-quadruplex/ligand. So far, studies on the binding stoichiometry of ZnTMPyP4 in complex with the three G-quadruplexes are limited. For example, only one article was found to report the stoichiometry of the binding of ZnTMPyP4 toward AG_3_(T_2_AG_3_)_3_ by using continuous variation analysis (Job plots)^22^. To determine the binding stoichiometry, we performed a series of triplet dynamics experiments under different molar ratios of [G-quadruplex]/[ligand], as those for 6:1. The triplet decay curves are displayed in Figure S5 and the fitted percentages of binding modes are plotted in Fig. 6 and listed in Table S1.

When the [AG_3_(T_2_AG_3_)_3_]/[ZnTMPyP4] molar ratio varies from 4:1 to 2:1, 1:1, and 1:2, all decay curves are biexponential and the fitted lifetime components (τ_1_, τ_2_) show the similar values to the case of 6:1 (Fig. 6(a)), except the relative percentage of these two component changes slightly. The two lifetime components correspond to end-stacking mode and intercalation mode, which is consistent with the results in the case of 6:1. This indicates that all the ZnTMPyP4 can be bound to AG_3_(T_2_AG_3_)_3_ although its concentration is two times larger than [AG_3_(T_2_AG_3_)_3_] at the molar ratio of 1:2. Interestingly, at the molar ratio of 1:3, that is, [ZnTMPyP4] is three times larger than [AG_3_(T_2_AG_3_)_3_], three lifetime components including τ_1_, τ_2_, and the free ZnTMPyP4 lifetime (2.9 μs) are required to obtain a reasonable fitting, indicating that excess ZnTMPyP4 molecules cannot be accommodated by AG_3_(T_2_AG_3_)_3_ and emerge as free species in the bulk solution. From these data, it can be concluded that up to two ZnTMPyP4 molecules can be bound with one AG_3_(T_2_AG_3_)_3_ and the molar ratio of 1:2 is the stoichiometry of the AG_3_(T_2_AG_3_)_3/_ZnTMPyP4 binding. The binding stoichiometry matches the earlier study, which reported the value of 1:2 by using continuous variation analysis (Job plots)^22^.

Similar analysis was carried out for (G_4_T_4_G_4_)2 and (TG_4_T)4. For (G_4_T_4_G_4_)2 (Fig. 6(b)), at molar ratio smaller than 1:1, free ZnTMPyP4 population already exists in the bulk solution, while in the range of the molar ratio from 1:1 to 6:1, all the ZnTMPyP4 are bound. Thus, the stoichiometry of (G_4_T_4_G_4_)2/ZnTMPyP4 is determined as 1:1. For (TG_4_T)4 (Fig. 6(c)), free ZnTMPyP4 population starts to emerge at the molar ratio of 1:3, thus the stoichiometry of the (TG_4_T)4_/_ZnTMPyP4 binding is 1:2.

**Figure 7:**
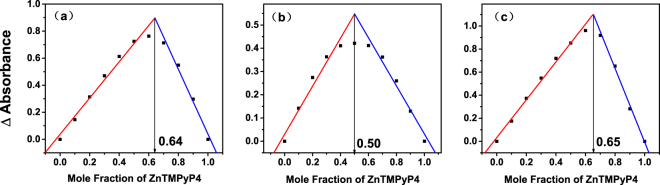
Job plots for the interaction of ZnTMPyP4 with the three G-quadruplexes: (**a**) AG_3_(T_2_AG_3_)_3_, (**b**) (G_4_T_4_G_4_)2 and (**c**) (TG_4_T)4. The fitted ratios were calculated and presented at each graph.

To further substantiate the binding stoichiometric values measured here, we performed the continuous variation analysis (Job plots). As shown in Fig. 7, the intersection points of two fitted lines for ZnTMPyP4 in complex with AG_3_(T_2_AG_3_)_3_, (G_4_T_4_G_4_)2 and (TG_4_T)4 are 0.64, 0.50, and 0.65, corresponding to the stoichiometry of the G-quadruplex_/_ZnTMPyP4 of 1:2, 1:1 and 1:2. These results confirm the binding stoichiometric values obtained by triplet decays, indicating that the transient spectral method here is feasible for the measurement of binding stoichiometry.

## Conclusion

In this work, we utilize triplet excited state as a sensitive reporter to investigate the binding of ZnTMPyP4 with three G-quadruplexes, i.e., the single-stranded AG_3_(T_2_AG_3_)_3_, the intermolecular double-stranded (G_4_T_4_G_4_)2, and the intermolecular four-stranded (TG_4_T)4. By monitoring the triplet excited state decay dynamics, it is observed that the bound ZnTMPyP4 exhibits biexponential decay dynamics with a slower lifetime component and a faster lifetime component markedly longer than that of free ZnTMPyP4. For the interaction of ZnTMPyP4 with AG_3_(T_2_AG_3_)_3_ or (G_4_T_4_G_4_)2, the slower lifetime component of about 31 μs and the faster lifetime component of about 5 μs are assigned to the intercalation binding mode and end-stacking binding mode, respectively. For ZnTMPyP4 interacting with (TG_4_T)4, the two lifetime components (~17 μs and ~5 μs) are ascribed to the partial intercalation mode and end-stacking mode. Further, the contributions of these coexisted binding modes are assessed on the basis of pre-exponential factors in the biexponential fitting equations. It is shown that for AG_3_(T_2_AG_3_)_3_ and (G_4_T_4_G_4_)2, the preferred binding mode is end-stacking (75% and 64%) whereas the percentages of intercalation are 25% and 36%. Compared with TMPyP4, the contributions of intercalation are markedly decreased, indicating that the steric hindrance of the axial water could prevent the intercalation of ZnTMPyP4. For (TG_4_T)4, the end-stacking mode accounts for 23%, and the partial intercalation mode accounts for 77%, which is similar to the case of TMPyP4. This is indicative of weak effect of the axial water on these two non-intercalative binding modes. By measuring triplet decay of ZnTMPyP4 under various [G-quadruplex]/[ZnTMPyP4] ratios, the triplet reporter method is extended to examine binding stoichiometry of G-quadruplex/ZnTMPyP4. The binding stoichiometric values are determined to be 1:2 for AG_3_(T_2_AG_3_)_3_, 1:1 for (G_4_T_4_G_4_)2, and 1:2 for (TG_4_T)4, which coincide with those obtained by the conventional method of continuous variation analysis. It is thus shown that the transient spectral method is feasible to determine binding stoichiometric ratios. These results allow a clear picture understanding of the G-quadruplex/ZnTMPyP4 interactions and are of fundamental importance for anticancer drug applications.

## Methods

### Materials

The DNA oligonucleotides TG_4_T, G_4_T_4_G_4_ and AG_3_(T_2_AG_3_)_3_ were purchased from the Sangon Biotech (Shanghai) Co.,Ltd. in the ULTRAPAGE-purified form. The absorbance in the UV-vis spectra was monitored at 260 nm to determine single-strand concentrations with the help of the corresponding extinction coefficients of 0.578 × 10^5^, 1.152 × 10^5^, and 2.285 × 10^5^ M^−1^cm^−1^ for TG_4_T, G_4_T_4_G_4_, and AG_3_(T_2_AG_3_)_3_, respectively^40^. The oligonucleotides were dissolved in a buffer solution containing 10 mM Tris-HCl, 1 mM EDTA, and 100 mM KCl for TG_4_T or 100 mM NaCl for AG_3_(T_2_AG_3_)_3_ and G_4_T_4_G_4_ at pH 7.5 to lead G-quadruplexes to form the targeted structures. The mixture was then first heated to 95 °C for 5 min before it was cooled down to room temperature with a cooling rate of 0.5 °C/min, and then incubated at 4 °C for 12 h.

The porphyrin derivative Zn(II) meso-Tetra (Nmethyl-4-pyridyl) Porphine (ZnTMPyP4) in the form of tetrachloride salt was purchased from J&K Scientific Ltd. and used as received. To prevent photodegradation, a 0.5 mM ZnTMPyP4 stock solution in ultrapure water (18.2 MΩ, purified by Millipore filtration) was stored in the dark. Freshly diluted ZnTMPyP4 buffer solution (10 mM Tris-HCl, 1 mM EDTA, and 100 mM KCl or 100 mM NaCl at pH 7.5) with 2 μM concentration was used in the experiment. The absorbance in the UV-vis spectra was monitored at 437 nm to determine ZnTMPyP4 concentrations with an extinction coefficient of 2.04 × 10^5^ M^−1^cm^−1^ ^19^.

### Sample preparation

Samples of G-quadruplexes and ZnTMPyP4 were mixed before each measurement, by adding G-quadruplex DNA stock solution and the freshly diluted ZnTMPyP4 buffer solution to related buffer solution, and the mixture was incubated at room temperature for 30 min to ensure good binding. To maintain zinc porphyrins under monomeric form without aggregation the concentration of ZnTMPyP4 was fixed at a low concentration of 2 μM in the mixed solution. All solutions subject to measurements were under the air-saturated conditions. All measurements were carried out in 1 cm path length quartz cuvettes at room temperature.

### Steady-State Spectral Measurements

Circular dichroism (CD) spectra of the four G-quadruplexes (10 μM) were recorded on a Spectro polarimeter (Jasco J-815) at room temperature. Under the condition of the measure range from 320 nm to 200 nm at a scan speed of 500 nm/min with a response time of 0.5 s, each of the G-quadruplex sample was measured three times, and the final spectra were the average of them. In addition, the averaged spectra should minus the background, that is, the spectrum from a blank sample containing only buffer. UV-vis absorption spectra of ZnTMPyP4 with the three G-quadruplexes (varied from zero to 12 μM) were recorded in the 350–600 nm range with a UV-vis spectrometer (model U-3010, Hitachi).

### Laser Flash Photolysis

Nanosecond time-resolved laser flash photolysis (LFP) was used to measure the transient UV-vis absorption spectra and triplet excited state decay dynamics that has been described previously^28^. Briefly speaking, the instrument comprises an Edinburgh LP920 spectrometer (Edinburgh Instrument Ltd.) combined with an Nd:YAG laser (Surelite, Continuum Inc.). The excitation wavelength is 355 nm laser pulse from Q-switched Nd:YAG laser (1 Hz, fwhm ≈ 7 ns, 10 mJ/pulse). The analyzing light was from a 450 W pulsed xenon lamp. To analyze transient absorption spectra, a monochromator equipped with a photomultiplier was used to collect the spectral with a range from 350 to 600 nm. The data were transferred to a personal computer after the signals from the photomultiplier were displayed and recorded as a function of time on a 100 MHz (1.25 Gs/s sampling rate) oscilloscope (Tektronix, TDS 3012 C). Data were analyzed by the online software of the LP920 spectrophotometer. The fitting quality was judged by weighted residuals and reduced χ^2^ value.

### Continuous Variation Analysis

A series of solution with varying mole fraction of ZnTMPyP4 and three G-quadruplexes but the same sum concentration (10 µM) were used for this experiment, while the ZnTMPyP4 solutions with corresponding concentrations were used as reference. Absorption difference spectra were collected from 350 to 600 nm with a 1 cm path-length quartz cell. For each spectrum collected, the porphyrin solution without an oligonucleotide was placed in the reference compartment and the corresponding ZnTMPyP4 solution with an oligonucleotide was placed in the sample compartment. The difference in the maximum and minimum absorbance values was plotted versus the ZnTMPyP4 mole fraction to generate a Job plot^13, 22^.

### Gel Electrophoresis

The molecular size of G-quadruplexes was visualized by nondenaturing polyacrylamide gel electrophoresis (PAGE). Sample was loaded on 18% polyacrylamide gels supplemented with K^+^ buffer (pH = 7.5) and run at 26 °C. 40% (v/v) sucrose was added before loading. The gels were revealed by silver staining^41^.

## Electronic supplementary material

Supporting Information
