# Supplementary material for: Interaction between G-Quadruplex and Zinc Cationic Porphyrin: The Role of the Axial Water
Source: Sci Rep. 2017 Sep 8;7:10951. doi: 10.1038/s41598-017-11413-8 (PMC5591184; doi:10.1038/s41598-017-11413-8)
Supplement: Supplementary file 1 — Supporting Information [file 41598_2017_11413_MOESM1_ESM.doc]

Supporting Information

**Interaction between G-Quadruplex and Zinc Cationic Porphyrin: The Role of the Axial Water**

Xiangzi Yao^1, 3^, Di Song^1^,*, Tingxiao Qin^1, 3^, Chunfan Yang^2^, Ze Yu^2^, Xiaohong Li^2^, Kunhui Liu^2^, and Hongmei Su^1, 2, 3^,*

^[1]^ *Beijing National Laboratory for Molecular Sciences (BNLMS), Institute of Chemistry, Chinese Academy of Sciences*

*Beijing, 100190, China*

^[2]^ *College of Chemistry, Beijing Normal University, Beijing, 100875, China*

^[3]^ *University of Chinese Academy of Sciences, Beijing, 100049, China*

*Corresponding author e-mail: [hongmei@bnu.edu.cn](mailto:hongmei@bnu.edu.cn); [songdi@iccas.ac.cn](mailto:hongmei@iccas.ac.cn)


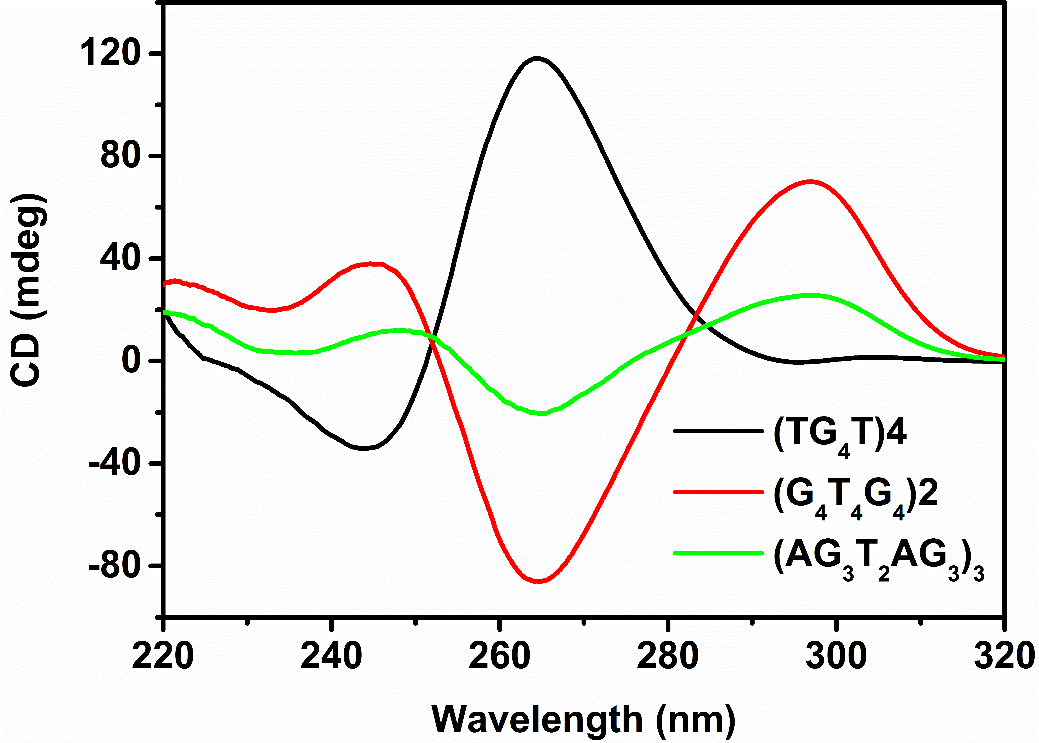


**Figure S1.** CD spectra of the three G-quadruplex DNAs (10 μM). (TG_4_T)4 were prepared in K^+^ buffer (10 mM Tris-HCl, 1 mM EDTA, and 100 mM KCl, pH=7.5), while (G_4_T_4_G_4_)2 and AG_3_(T_2_AG_3_)_3_ were prepared in Na^+^ buffer (10 mM Tris-HCl, 1 mM EDTA, and 100 mM NaCl, pH=7.5).

**Figure S2.** (a)-(c) Possible binding modes of ZnTMPyP4 with AG_3_(T_2_AG_3_)_3_, (G_4_T_4_G_4_)2 and (TG_4_T)4, respectively. It is noted that end-stacking mode within loop regions or the thymine bases at two ends are both possible, and only one pattern is depicted here.

**
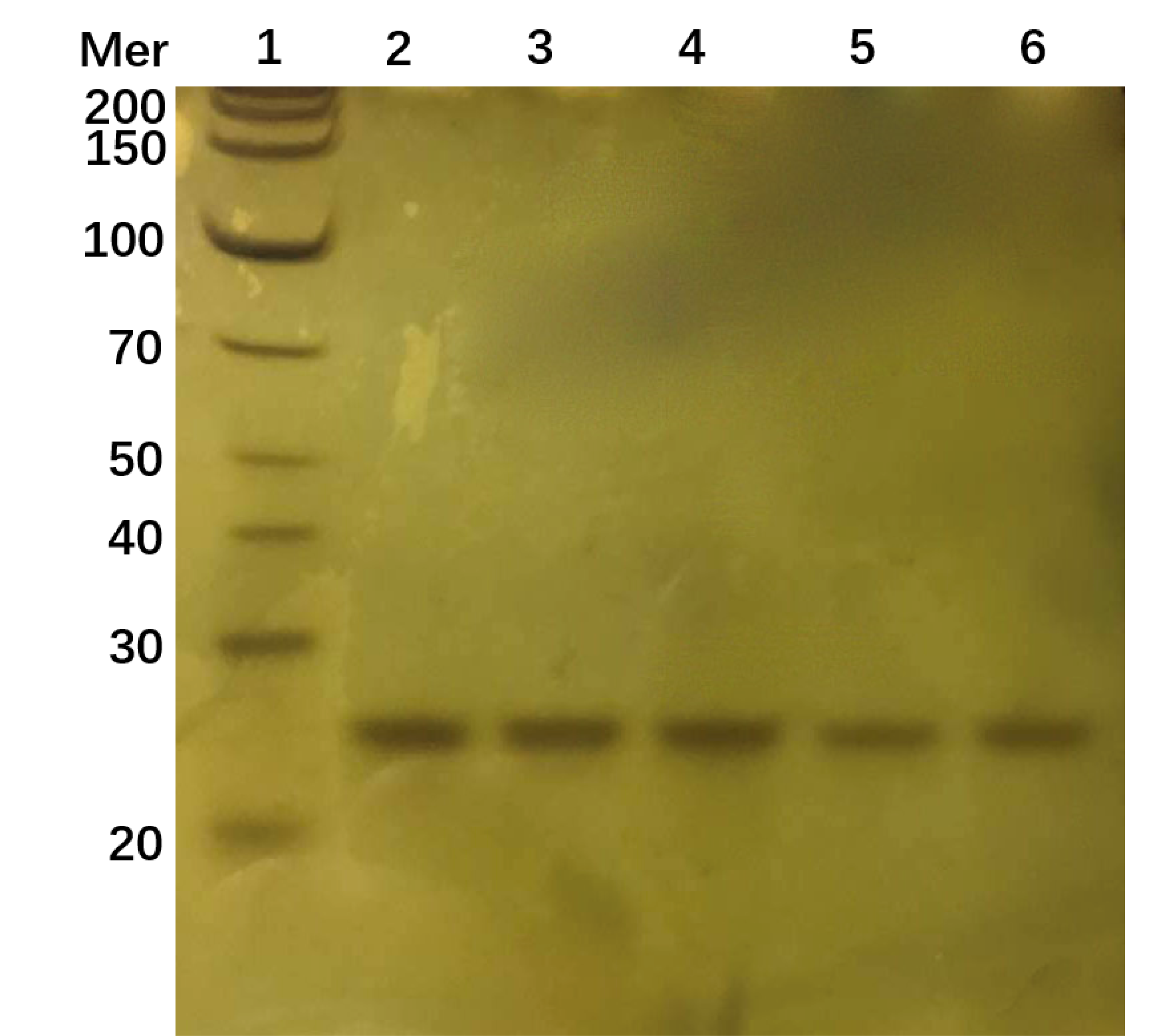
**

**Figure S3.** The origin full-length polyacrylamide gel analysis of (TG_4_T)4 in the presence of K^+^. Lane 1: DNA marker 10-mer ladder; Lane 2: 1.5 μM (TG_4_T)4; Lane 3: 1.5 μM (TG_4_T)4 with 0.25 μM TMPyP4; Lane 4: 1.5 μM (TG_4_T)4 with 0.25 μM ZnTMPyP4; Lane 5:1.5 μM (TG_4_T)4 with 3 μM TMPyP4; and Lane 6: 1.5 μM (TG_4_T)4 with 3 μM TMPyP4.


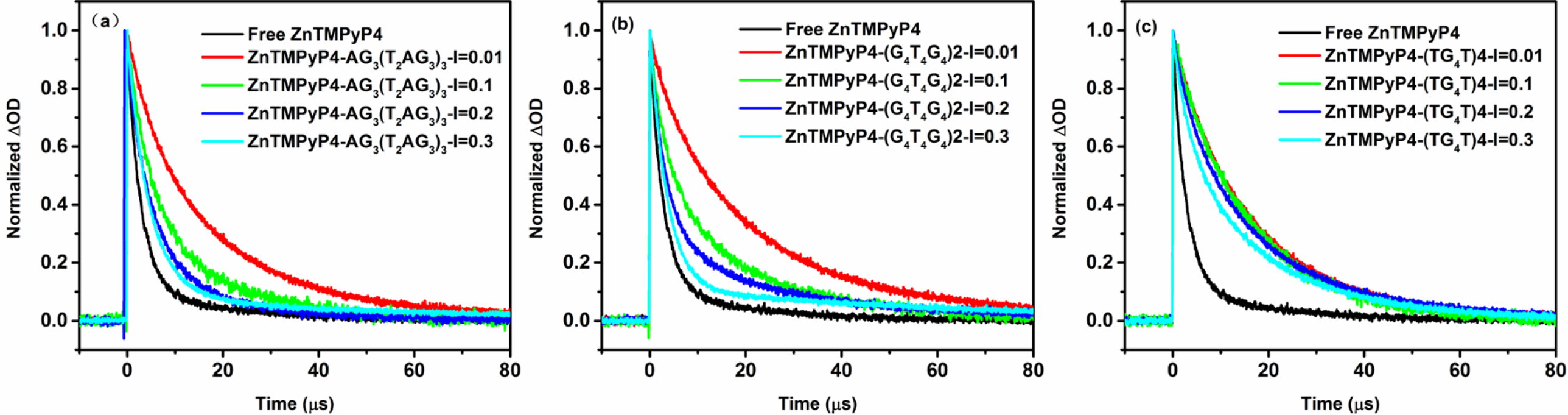


**Figure S4.** Normalized triplet decay signals after laser flash photolysis of ZnTMPyP4 (2 μM) upon 355 nm excitation in the presence of G-quadruplex (a) AG_3_(T_2_AG_3_)_3_, (b) (G_4_T_4_G_4_)2, (c) (TG_4_T)4 at different ionic strength.


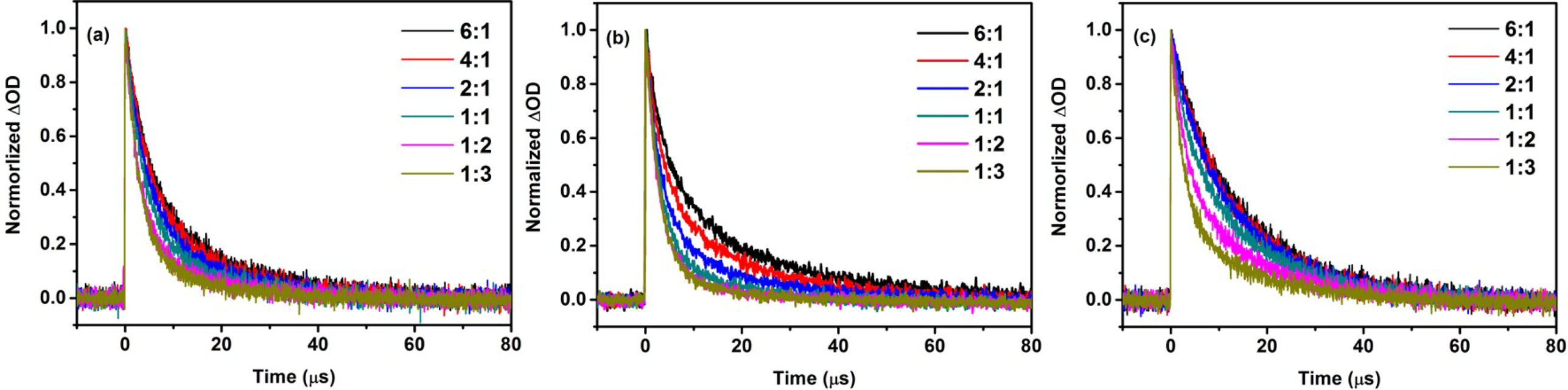


**Figure S5.** Normalized triplet decay signals after laser flash photolysis of ZnTMPyP4 (2 μM) upon 355 nm excitation in the presence of G-quadruplex (a) AG_3_(T_2_AG_3_)_3_, (b) (G_4_T_4_G_4_)2, (c) (TG_4_T)4. Results for the serial [G-quadruplex]/[ZnTMPyP4] molar ratios (6:1, 4:1, 2:1, 1:1, 1:2, and 1:3) are displayed with different colors.

**Table S1** The population percentage of free ZnTMPyP4 and bound ZnTMPyP4 in the intercalation mode, in the end-stacking mode, and in the partial intercalation mode, which are obtained for different [G-quadruplex]/[ZnTMPyP4] molar rations of 6:1, 4:1, 2:1, 1:1, 1:2 and 1:3: (a) [AG_3_(T_2_AG_3_)_3_]/[ZnTMPyP4], (b) [(G_4_T_4_G_4_)2]/[ZnTMPyP4] and (c) [(TG_4_T)4]/[ZnTMPyP4].

(a)

| [AG_3_(T_2_AG_3_)_3_]/[ZnTMPyP4] Ratio | Binding modes | | |
| --- | --- | --- | --- |
|  | end-stacking | intercalation | free ZnTMPyP4 |
| 6:1 | 75% | 25% | N/A |
| 4:1 | 77% | 23% | N/A |
| 2:1 | 83% | 17% | N/A |
| 1:1 | 87% | 13% | N/A |
| 1:2 | 96% | 4% | N/A |
| 1:3 | 51% | N/A | 49% |

(b)

| [(G_4_T_4_G_4_)2]/[ZnTMPyP4]  Ratio | Binding modes | | |
| --- | --- | --- | --- |
|  | end-stacking | intercalation | free ZnTMPyP4 |
| 6:1 | 64% | 36% | N/A |
| 4:1 | 72% | 28% | N/A |
| 2:1 | 87% | 13% | N/A |
| 1:1 | 100% | N/A | N/A |
| 1:2 | 30% | N/A | 70% |
| 1:3 | 21% | N/A | 79% |

(c)

| [(TG_4_T)4]/[ZnTMPyP4]  Ratio | Binding modes | | |
| --- | --- | --- | --- |
|  | end-stacking | partial intercalation | free ZnTMPyP4 |
| 6:1 | 23% | 77% | N/A |
| 4:1 | 28% | 72% | N/A |
| 2:1 | 36% | 64% | N/A |
| 1:1 | 45% | 55% | N/A |
| 1:2 | 63% | 37% | N/A |
| 1:3 | 38% | 27% | 25% |
